# Evaluation of dynamic recurrence risk for locally advanced gastric cancer in the clinical setting of adjuvant chemotherapy: a real-world study with IPTW-based conditional recurrence analysis

**DOI:** 10.1186/s12885-023-11143-3

**Published:** 2023-10-12

**Authors:** Dong Wu, Jun Lu, Zhen Xue, Qing Zhong, Bin-bin Xu, Hua-Long Zheng, Guo-sheng Lin, Li-li Shen, Jia Lin, Jiao-bao Huang, Davit Hakobyan, Ping Li, Jia-Bin Wang, Jian-Xian Lin, Qi-Yue Chen, Long-Long Cao, Jian-Wei Xie, Chang-Ming Huang, Chao-Hui Zheng

**Affiliations:** 1 Department of Gastric Surgery, Fujian Medical University Union Hospital, No.29 Xinquan Road, FuzhouFujian Province, 350001 China; 2 Department of General Surgery, Fujian Medical University Union Hospital, Fuzhou, China; 3 Key Laboratory of Ministry of Education of Gastrointestinal Cancer, Fujian Medical University, Fuzhou, China; 4 Fujian Key Laboratory of Tumor Microbiology, Fujian Medical University, Fuzhou, China

**Keywords:** Gastric cancer, Adjuvant chemotherapy, Conditional survival, Recurrence

## Abstract

**Background:**

The long-term dynamic recurrence hazard of locally advanced gastric cancer (LAGC) in the clinical setting of adjuvant chemotherapy (ACT) remains unclear.

**Purpose:**

This study aimed to investigate the dynamic recurrence risk of LAGC in patients who received ACT or not.

**Methods:**

The study assessed data from patients with LAGC who underwent radical gastrectomy between January, 2010 and October, 2015. Inverse probability of treatment weighting (IPTW) was performed to reduce selection bias between the ACT and observational (OBS) groups. Conditional recurrence-free survival (cRFS) and restricted mean survival time (RMST) were used to assess the survival differences.

**Results:**

In total, 1,661 LAGC patients were included (ACT group, *n* = 1,236 and OBS group, *n* = 425). The recurrence hazard gradually declined; in contrast, cRFS increased with RFS already accrued. Following IPTW adjustment, the cRFS rates were higher in the ACT group than those in the OBS group for patients at baseline or with accrued RFS of 1 and 2 years (*p*˂0.05). However, the cRFS rates of the ACT group were comparable with those of the OBS group for patients with accrued RFS of 3 or more years (*p* > 0.05). Likewise, the 5-year △RMST between the ACT and OBS groups demonstrated a similar trend. Moreover, the hematological metastasis rate of the ACT group was significantly lower than that of the OBS group for patients at baseline or with accrued RFS of 1 and 2 years, respectively (*p*˂0.05).

**Conclusions:**

Although ACT could provide substantial benefits for patients with LAGC, the differences in recurrence hazard between the ACT and OBS groups may attenuate over time, which could help guide surveillance and alleviate patients’ anxiety. Further prospective large-scale studies are warranted.

**Supplementary Information:**

The online version contains supplementary material available at 10.1186/s12885-023-11143-3.

## Introduction

Gastric cancer is the fifth most common malignancy and the third leading cause of cancer-related deaths worldwide [1]. Recent advances in multidisciplinary approaches have prolonged the life of cancer patients. Thus, the long-term dynamic recurrence of cancer survivors triggers many concerns [2].

Given many prospective trials [3–5] confirmed the survival benefits of adjuvant chemotherapy (ACT), ACT has become one of the standard treatments for locally advanced gastric cancer (LAGC). However, above studies assessed the long-term survival of patients using actual or actuarial survival data, which have limited informativeness from the dynamic and long-term standpoint, especially in patients with a poor prognosis in the early period. Besides, scholars have developed various prediction models to stratify patients into different risks, which helped optimize the follow-up strategies and select patients who can benefit from ACT [6–8]. Whereas, the conventional “static” prediction model does not account for the effect of time after surgery, generates more inaccurate information as time from surgery elapses, and tends to underestimate the survival probability of long-term survivors [9–11]. Furthermore, with the improvement of the prognosis of cancer patients, more and more cancer survivors may face the fear of cancer recurrence in the remaining lifespan, which would impair psychological function and quality of life, especially for patients with poor pathological grades [12, 13].

**Fig. 1:**
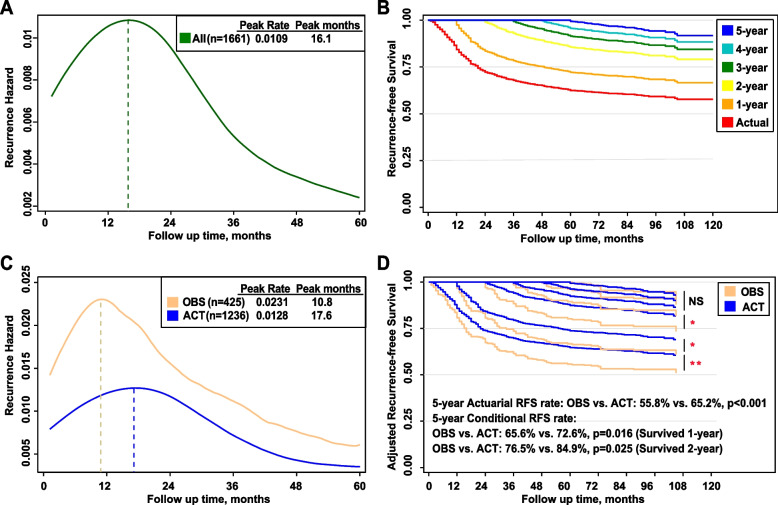
Dynamic recurrence hazard curves (**A**), and stratified by the ACT and OBS groups (**C**), Kaplan–Meier curve of conditional recurrence-free survival (cRFS) (**B**), and stratified by the ACT and OBS groups (**D**) * indicates *P* ˂ 0.05; ** indicates *P* ˂ 0.01. NS indicates not significant

Thus, a new concept, conditional recurrence-free survival (cRFS) was put forward, which was derived from the conception of conditional probability (S-Fig. 1). cRFS represents the probability of recurrence-free survival (RFS) in the subgroup of patients who have already accrued RFS for a given time after surgery. Compared with traditional RFS, cRFS provides more accurate information regarding the dynamic changes of recurrence risk, which is widely used in other malignancies, including rectal cancer, liver cancer, bladder cancer, and pancreatic neuroendocrine tumors [14–17]. In clinical practice, cRFS is a promising indicator to help clinicians develop surveillance schedules and modify them timely according to the dynamic hazard. Most patients with LAGC would be informed of a poor prognosis according to pathological reports. Since then, the fear and anxiety of cancer recurrence may accompany them in their whole lives. Understanding the possibility of continued RFS over time (longer accrued RFS, higher additional survival) will help alleviate the anxiety and improve the quality of life of patients with LAGC, especially those with a poor prognosis.

So far, the dynamic changes in recurrence hazard of the ACT and observational (OBS) groups have not been reported. Therefore, our study aimed to utilize cRFS and restricted mean survival time (RMST) as prognostic indicators to reappraise the long-term dynamic risk of patients with ACT or not, which can help clinical physicians provide effective interventions and appropriate psychological supports to ease the disease burden and psychological distress of patients.

## Materials and methods

### Patients

Utilizing a prospective gastric cancer database, we identified 2,417 patients with primary gastric adenocarcinoma who underwent curative gastrectomy at the Fujian Medical University Union Hospital between January, 2010 and October, 2015.

The inclusion criteria involved (1) Eastern Cooperative Oncology Group (ECOG) scores of 0 or 1; (2) pathologically confirmed LAGC (pstage II and III, except pT4b); (3) D2 lymph node dissection of gastric cancer; (4) no invasion of adjacent organs or distant metastasis (e.g. pancreas, spleen, liver, and colon) intraoperatively or postoperatively.

**Fig. 2:**
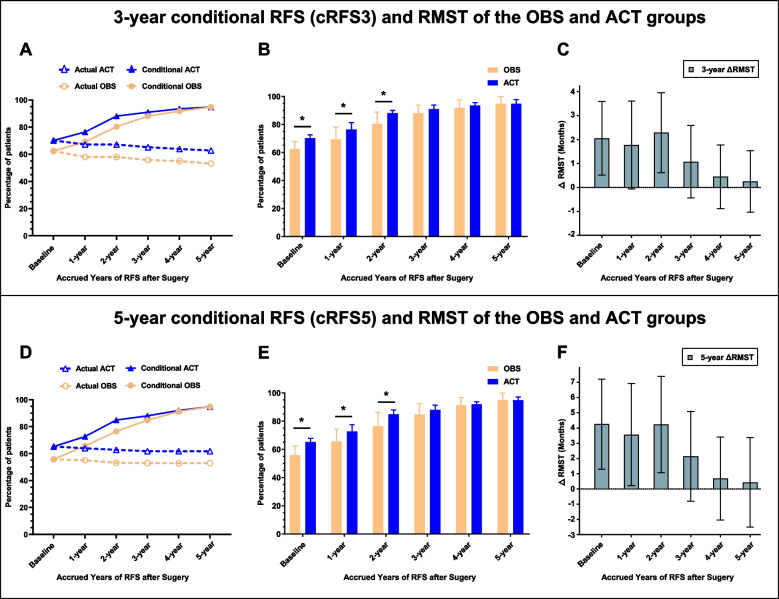
Comparison of actual 3-year recurrence-free survival (RFS), cRFS3 and 3-year △RMST between the ACT and OBS groups following IPTW adjustment (**A**-**C**). Comparison of actual 5-year recurrence-free survival (RFS), cRFS5 and 5-year △RMST between the ACT and OBS groups following IPTW adjustment (**D**-**F**). Notes: △RMST indicates the differences in RMST between the ACT and OBS groups. For example, 5-year △RMST indicates that after X years of recurrence-free survival (RFS), the mean RFS in the ACT group was n months longer than that in the OBS group within 5 years. * indicates *P* ˂ 0.05

The exclusion criteria involved (1) American Society of Anesthesiologists (ASA) grades exceed 2; (2) neuroendocrine or remnant gastric cancer; (3) history of preoperative chemotherapy; (4) palliative surgery; and (5) death within 1 month after surgery. Eventually, 1,661 patients were included as the primary cohort (S-Fig. 2). All patients were informed in detail about the advantages and disadvantages of laparoscopic and open gastrectomy before the surgery, and the patients consented to the surgical approaches.

### Cancer staging

LAGC was defined as the American Joint Committee on Cancer (AJCC) pathological stage II to III without T4b tumors (including stage II: T2N1-2M0, T3N0-1M0, and T4aN0M0 and stage III: T2N3M0, T3N2-3M0, and T4aN1-3M0). The 8^th^ AJCC Cancer Staging Manual was used to further categorization [18, 19].

### Definitions

ACT was recommended for all patients with LAGC [16]. The 5-fluorouracil-based regimen, S-1 plus oxaliplatin (SOX), was routinely administered at our center.

RFS was defined as the time from surgical resection to initial disease relapse or completion of the last follow-up. Recurrence was defined as the initial disease recurrence after surgery, and it was categorized as locoregional, peritoneal, distant, or multiple sites. Recurrence at ≥ 2 sites was defined as multiple sites of recurrence and not multiple recurrences at the same site. Locoregional recurrence included masses in the gastric bed, D2 lymphadenectomy nodal stations, or anastomotic recurrences. Peritoneal recurrence was defined as positive cytology in the ascitic fluid or as the convincing presence of peritoneal nodules on cross-sectional imaging, as documented in the radiology report. Distant metastases were further defined according to the specific organs and distant lymph nodes. Diseases involving the cervical lymph or abdominal nodes beyond the upper retroperitoneum were considered distant metastasis. Mediastinal lymph node recurrences were considered locoregional for gastroesophageal junction tumors and distant for all other tumors. Hematological metastasis was defined as specific organs involved, including the liver, lung, bone, and others. Tumors involving the ovaries were considered peritoneal recurrences and were classified as Krukenberg tumors [20]. All recurrences were documented using pathological diagnosis and/or radiologic imaging. Radiologic proof of recurrence was specifically reviewed in the context of the clinical situation and typically required sequential imaging and demonstrating the progression of metastatic lesions.

### Follow-up schedule

The median follow-up period was 55 months. Follow-up was conducted according to the Japanese gastric cancer treatment guidelines 2014 (ver. 4) [18]. Generally, follow-up was performed every 3 months for the first 2 years and every 6 months thereafter for 5 years for patients with LAGC. Follow-up interventions consisted of physical examination, laboratory tests, chest radiography, abdominal ultrasound, abdominopelvic computed tomography (CT) imaging, and annual gastroscopy; further magnetic resonance imaging (MRI) and positron emission tomography (PET)-CT were performed, if necessary. Recurrence was diagnosed based on positive radiological evidence. Patients were followed-up until death or the cut-off date of April, 2020. Patients who were lost to follow-up or died following the operation were treated as censored cases.

### Statistical analysis

Continuous variables were presented as mean ± standard deviation, while categorical variables were presented as numbers. The distributions of each continuous and categorical variable were compared using student’s t-test, χ^2^ test, or categorical Fisher exact test, as appropriate. The Cox proportional hazard models were used to estimate the hazard ratios (HRs) for RFS. Monthly hazard rates were estimated at monthly intervals using a kernel Epanechnikov smoothing method. The hazard function conveys information about the risk of an event at time t, not for the entire cohort, but only for those patients remaining at risk at time t. In other words, the hazard function evaluates an instantaneous conditional hazard rate [21].

### Inverse probability of treatment weighting

Baseline characteristics of LAGC patients who underwent ACT (ACT group) were compared with those who did not undergo ACT following surgery (OBS group). The balance in covariates was assessed using the standardized mean difference (SMD) approach [22]. Factors with an imbalance between the two groups were defined as SMD > 0.1. Multivariate logistic regression models were used to estimate the association of the included covariates with ACT. Differences in baseline covariates between both groups were adjusted using the inverse probability of treatment weighting (IPTW) method [23]. The IPTW approach mimics a situation in which treatments were randomly allocated to individuals through weighing. To get the propensity score, we included the following factors associated with and without ACT in the models: age, sex, Charlson comorbidity index, ASA, pathological T (pT) stage, pathological N (pN) stage, Clavien–Dindo grade, histological grade, and tumor size. Suppose that there are N participants in a data set, with n1 participants who received the ACT and n0 participants who did not; *N* = n0 + n1. The probability of the ACT group is *p* = n1/N, and the probability of the OBS group is 1—p. The propensity score was estimated with the above model named π_i_. The weights of individuals were calculated by the following algorithm: The weights of patients in the ACT group were defined as W = p/π_i_, and the weights of patients in the OBS group were defined as W = (1-p)/(1-π_i_). The adjusted Kaplan–Meier curves and log-rank test based on IPTW were computed to compare the cRFS rates and RMST between both groups.

### Conditional survival

Conditional survival was calculated as CPS (*n*) = S(x + n) / S(x), (x < y), where y is the probability of surviving for y years, given that the person has survived for x years, as previously described by Zabor et al. [24]. Actual recurrence data were used for these calculations. cRFS were calculated for 3 and 5 additional years (cRFS3 and cRFS5) at each time point to obtain concise, clinically significant data. For instance, the cRFS3 of patients at 1 year can be calculated as the RFS rate at (1 + 3 =) 4 years (RFS4) divided by the RFS rate at 1 year (RFS1) (cRFS3 = RFS4/RFS1), and represents the percentage of patients who have been recurrence-free at 1 year that can be expected to remain recurrence-free after an additional 3 years.

### RMST

RMST represents the average life expectancy in a period. The difference in RMST (△RMST) helps compare the efficacies of different treatments, which has been widely used in the long-term evaluation of esophageal, renal, and breast cancers [25–27]. Theoretically, RMST is the area under the survival curve from 0 to t* and is interpreted as the life expectancy between randomization (t = 0) and a particular time horizon (t*) [28, 29]. In this study, RMST was calculated within the 3-year or 5-year time horizon (t*) and adjusted using the IPTW approach as mentioned above. The △RMST was defined as the difference in RMST between the ACT and OBS groups, which was tested using the log-rank test or the Grambsch–Therneau test [30].

All *p*-values were two-sided, and *p* < 0.05 was considered statistically significant and marked ‘ *’ in the figures. All statistical analyses were performed using R version 3.6.2 (R Foundation for Statistical Computing, Vienna, Austria).

## Results

### Overview of the primary cohort

#### Clinicopathological characteristics of the primary cohort

S-Table 1 shows the clinicopathological characteristics of the primary cohort. There were 1,661 patients with LAGC who underwent radical gastrectomy between 2010 and 2015, including 1,230 (74.1%) men and 431 (25.9%) women. ACT was administered to 1,236 (74.4%) patients.

#### Dynamic recurrence hazard of the primary cohort

Recurrence hazard peaked at 16.1 months with a peak rate of 0.0109 and then gradually decreased (Fig. 1A). cRFS rates gradually increased with RFS already accrued (Fig. 1B). The 3-year and 5-year actual RFS rates were 66.8% and 62.0%, respectively. The 3-year cRFS rates (cRFS3, achieving additional 3-year RFS) were 76.0%, 85.8%, 91.2%, 93.7%, and 95.4% for patients with LAGC with accrued RFS of 1 year, 2 years, 3 years, 4 years, and 5 years, respectively. Likewise, the 5-year cRFS rates (cRFS5, achieving additional 5-year RFS) were 72.2%, 83.1%, 88.5%, 89.8%, and 92.9% for patients with LAGC with accrued RFS of 1 year, 2 years, 3 years, 4 years, and 5 years, respectively (S-Table 2).

#### Univariate and multivariate Cox regression at baseline

Based on univariate analysis, ACT, surgical approach, age, tumor location, tumor size, perineural invasion, lymphovascular invasion, histological grade, pT stage, pN stage, and Clavien–Dindo grade were associated with RFS in patients with LAGC at baseline (S-Table 3). Multivariate analysis showed that ACT was an independent prognostic factor (HR, 0.67; 95% confidence interval [CI], 0.56–0.81; *p* < 0.001), alongside pT stage, pN stage, and tumor size (*p* < 0.05) (Table 1).

**Table 1:** Time-dependent multivariate analysis of the Recurrence-free Survival for LAGC patients

Clinical characteristics	Baseline			1-year			2-year			3-year			4-year		
	***N* = 1661, E = 605**			***N* = 1370, E = 388**			***N* = 1110, E = 176**			***N* = 1009, E = 106**			***N* = 917, E = 62**		
	**HR**	**95% CI**	***p* value**	**HR**	**95% CI**	***p* value**	**HR**	**95% CI**	***p* value**	**HR**	**95% CI**	***p* value**	**HR**	**95% CI**	***p* value**
**Age**															
< 65 years	Ref			Ref											
≥ 65 years	1.13	0.95–1.34	0.175	1.21	0.97–1.52	0.086									
**Histology**															
High/ moderate differentiated	Ref														
Low differentiated/ undifferentiated	0.84	0.70–1.01	0.070												
**Pathologic T Stage**															
T1	Ref			Ref											
T2	0.77	0.34–1.75	0.536	0.75	0.26–2.12	0.585									
T3	1.27	0.62–2.61	0.518	1.45	0.58–3.61	0.423									
T4a	2.12	1.03–4.34	**0.040**	2.34	0.94–5.79	0.067									
**Pathologic N Stage**															
N0	Ref			Ref			Ref			Ref			Ref		
N1	1.94	1.25–3.01	**0.003**	1.85	1.12–3.06	**0.016**	1.15	0.60–2.22	0.673	1.5	0.61–3.68	0.376	1.88	0.64–5.51	0.248
N2	2.79	1.87–4.15	** < 0.001**	2.70	1.71–4.25	** < 0.001**	2.37	1.35–4.15	**0.003**	2.4	1.13–5.10	**0.023**	2.69	0.98–7.34	0.054
N3a	5.14	3.51–7.52	** < 0.001**	4.19	2.69–6.53	** < 0.001**	3.6	2.07–6.25	** < 0.001**	3.59	1.62–7.99	**0.002**	4.46	1.66–11.98	**0.003**
N3b	9.33	6.23–13.96	** < 0.001**	7.61	4.65–12.44	** < 0.001**	5	2.57–9.74	** < 0.001**	4.56	1.84–11.32	**0.001**	5.89	1.94–17.89	**0.002**
**Tumor Location**															
Lower	Ref			Ref			Ref								
Middle	0.96	0.77–1.20	0.732	1.01	0.75–1.35	0.961	1.17	0.77–1.79	0.458						
Upper	0.93	0.75–1.15	0.508	0.95	0.72–1.26	0.722	1.13	0.76–1.69	0.534						
Mixed	1.03	0.81–1.31	0.825	1.11	0.80–1.53	0.525	1.35	0.83–2.19	0.229						
**Tumor Size**															
< 50 mm	Ref			Ref			Ref			Ref			Ref		
≥ 50 mm	1.27	1.06–1.52	**0.009**	1.19	0.95–1.50	0.136	1.18	0.85–1.64	0.323	1.25	0.77–2.05	0.367	1.46	0.86–2.49	0.161
**PNI**															
No	Ref			Ref						Ref					
Yes	1.1	0.92–1.31	0.292	1.29	1.02–1.62	**0.034**				1.38	0.84–2.26	0.204			
**LVI**															
No	Ref			Ref											
Yes	1.06	0.88–1.26	0.549	0.94	0.74–1.19	0.616									
**Surgical approach**															
OG	Ref			Ref											
LG	0.83	0.67–1.02	0.069	0.72	0.55–0.94	**0.016**									
**The number of examined lymph nodes**				1	0.99–1.00	0.404	1	0.99–1.01	0.704						
**Postoperative complication**															
˂III	Ref														
≥ III	1.17	0.80–1.72	0.413												
**Adjuvant Chemotherapy**															
No	Ref			Ref			Ref			Ref					
Yes	0.67	0.56–0.81	** < 0.001**	0.7	0.55–0.90	**0.005**	0.63	0.45–0.90	**0.011**	0.64	0.38–1.08	0.097			

#### Comparison of clinicopathological characteristics between the ACT and OBS groups before and after IPTW

Table 2 shows the clinicopathological characteristics before and after IPTW. After adjusting for age, sex, Charlson comorbidity index, ASA, pT stage, pN stage, Clavien–Dindo grade, histological grade, and tumor size, no significant differences were observed in baseline characteristics between the ACT and OBS groups after IPTW (all SMD ˂0.1).

**Table 2:** Clinicopathological characteristics before and after IPTW in the baseline population

**Clinical characteristics**	**Before IPTW**			**After IPTW**		
	**OBS (*n* = 425)**	**ACT (*n* = 1236)**	**SMD**	**OBS (*n* = 422.3)**	**ACT (*n* = 1246.9)**	**SMD**
**Age n (%)**			0.585			0.013
< 65 years	175 (41.2%)	854 (69.1%)		261.4(61.9%)	763.9(61.3%)	
≥ 65 years	250 (58.8%)	382 (30.9%)		160.9 (38.1%)	483.0 (38.7%)	
**BMI n (%)**			0.090			0.099
< 25 kg/m^2^	326 (76.7%)	902 (73.0%)		326.3 (77.3%)	911.7 (73.1%)	
≥ 25 kg/m^2^	88 (20.7%)	302 (24.4%)		85.3 (20.2%)	302.4 (24.3%)	
unknown	11 (2.6%)	32 (2.6%)		10.7 ( 2.5%)	32.7 ( 2.6%)	
**Sex n (%)**			0.147			0.023
Male	294 (69.2%)	936 (75.7%)		316.6(75.0%)	922.1(73.9%)	
Female	131 (30.8%)	300 (24.3%)		105.7 (25.0%)	324.8 (26.1%)	
**Comorbidity n (%)**			0.174			0.005
No	282 (66.4%)	918 (74.3%)		301.4(71.4%)	893(71.6%)	
Yes	143 (33.6%)	318 (25.7%)		120.9 (28.6%)	353.9 (28.4%)	
**ASA n (%)**			0.177			0.003
I	214 (50.4%)	731 (59.1%)		234.9 (55.6%)	695.5 (55.8%)	
II	211 (49.6%)	505 (40.9%)		187.5 (44.4%)	551.4 (44.2%)	
**ECOG scores n (%)**			0.142			0.018
0	39 (9.2%)	169 (13.7%)		49.7(11.8%)	154(12.3%)	
1	386 (90.8%)	1067 (86.3%)		372.6 (88.2%)	1092.9 (87.7%)	
**Oncological characteristics**						
**Histology n (%)**			0.278			0.079
High/ moderate differentiated	167 (39.3%)	417 (33.7%)		142.5 (33.7%)	432.6 (34.7%)	
Low differentiated/ undifferentiated	242 (56.9%)	814 (65.9%)		274.3 (65.0%)	785.7 (63.0%)	
G_x_	16 (3.8%)	5 (0.4%)		5.5 ( 1.3%)	28.6 ( 2.3%)	
**Pathologic T Stage n (%)**			0.142			0.059
T1	8 (1.9%)	29 (2.3%)		8.5 ( 2.0%)	26.7 ( 2.1%)	
T2	23 (5.4%)	110 (8.9%)		30.2 ( 7.2%)	97.9 ( 7.8%)	
T3	190 (44.7%)	540 (43.7%)		176.5 (41.8%)	537.4 (43.1%)	
T4a	204 (48.0%)	557 (45.1%)		207.1 (49.0%)	588.8 (46.9%)	
**Pathologic N Stage n (%)**			0.239			0.055
N0	82 (19.3%)	173 (14.0%)		70.5 (16.7%)	192.0 (15.4%)	
N1	62 (14.6%)	221 (17.9%)		67.4 (16.0%)	216.5 (17.4%)	
N2	88 (20.7%)	303 (24.5%)		100.2 (23.7%)	287.6 (23.1%)	
N3a	142 (33.4%)	336 (27.2%)		118.3 (28.0%)	362.6 (29.1%)	
N3b	51 (12.0%)	203 (16.4%)		65.9 (15.6%)	188.1 (15.1%)	
**Pathological TNM Stage of 8th AJCC n (%)**			0.146			0.082
IIa	73 (17.2%)	191 (15.5%)		70.9 (16.8%)	192.7 (15.5%)	
IIb	67 (15.8%)	201 (16.3%)		63.4 (15.0%)	199.6 (16.0%)	
IIIa	96 (22.6%)	320 (25.9%)		109.9 (26.0%)	307.1 (24.6%)	
IIIb	131 (30.8%)	319 (25.8%)		107.4 (25.4%)	354.2 (28.4%)	
IIIc	58 (13.6%)	205 (16.6%)		70.7 (16.7%)	193.2 (15.5%)	
**Tumor Location n (%)**			0.131			0.074
Lower	160 (37.6%)	479 (38.8%)		170.0 (40.3%)	472.9 (37.9%)	
Middle	76 (17.9%)	275 (22.2%)		81.7 (19.3%)	267.5 (21.5%)	
Upper	121 (28.5%)	309 (25.0%)		107.6 (25.5%)	336.0 (26.9%)	
Mixed	68 (16.0%)	173 (14.0%)		63.0 (14.9%)	170.5 (13.7%)	
**Tumor Size n (%)**			0.155			0.001
< 50 mm	168 (39.5%)	583 (47.2%)		189.3(44.8%)	558(44.7%)	
≥ 50 mm	257 (60.5%)	653 (52.8%)		233.0 (55.2%)	688.9 (55.3%)	
**PNI n (%)**			0.055			0.010
No	304 (71.5%)	853 (69.0%)		266.7(63.2%)	793.8(63.7%)	
Yes	121 (28.5%)	383 (31.0%)		155.6 (36.8%)	453.1 (36.3%)	
**LVI n (%)**			0.008			0.064
No	276 (64.9%)	798 (64.6%)		299.8(71.0%)	848.4(68%)	
Yes	149 (35.1%)	438 (35.4%)		122.5 (29.0%)	398.5 (32.0%)	
**Other characteristics**						
**Surgical approach n (%)**			0.044			0.003
OG	78 (18.4%)	206 (16.7%)		73.4(17.4%)	215.2(17.3%)	
LG	347 (81.6%)	1030 (83.3%)		348.9 (82.6%)	1031.7 (82.7%)	
**The number of examined lymph nodes (n)**	35.5 ± 14.0	37.4 ± 14.2	0.135	36.46 (14.10%)	36.98 (14.38%)	0.036
**Postoperative complication n (%)**			0.187			0.008
˂III	397 (93.4%)	1197 (96.8%)		311.5 (73.8%)	922.8 (74.0%)	
≥ III	28 (6.6%)	36 (2.9%)		109.9 (26.0%)	321.8 (25.8%)	
unknown	0 (0.0%)	3 (0.2%)		0.9 ( 0.2%)	2.2 ( 0.2%)	

*PNI* Perineural invasion, *LVI* Lymphovascular invasion, *ASA* American Society of Anesthesiologists, *ECOG* Eastern Cooperative Oncology, *ACT* adjuvant chemotherapy group, *OBS* observational group

#### Dynamic recurrence hazard and cRFS between the ACT and OBS groups after IPTW

Figure 1C shows the dynamic recurrence hazards of the ACT and OBS groups after IPTW. The recurrence hazard curve for the ACT group peaked at 17.6 months (peak hazard rate: 0.0128) and gradually decreased with a long hem to the right. While in the OBS group, the recurrence hazard curve increased more sharply than that of the ACT group and peaked at 10.8 months (peak hazard rate: 0.0231), followed by a decrease with a long hem to the right.

In general, cRFS rates gradually increased with RFS in the ACT and OBS groups. In patients at baseline or with accrued RFS of 1 and 2 years, cRFS rates of the ACT group were significantly higher than those of the OBS group (log-rank *p*˂0.05) (Fig. 1D). In patients with accrued RFS of 3 or more years, the cRFS rates between both groups were comparable (log-rank *p* > 0.05) (Fig. 1D). More details of the actual RFS and cRFS between the ACT and OBS groups are illustrated in S-Table 4.

Figure 2 shows the actual RFS and dynamic changes in annual cRFS3 and cRFS5 rates within 5 years after surgery. As shown in Fig. 2A, the 3-year actual RFS and cRFS3 rates for the ACT and OBS groups were equal at baseline; however, contrary to the declining trend of the actual RFS curve, the cRFS3 rates increased with prolonged RFS. Moreover, with RFS annually accrued, the differences in cRFS3 between the ACT and OBS groups gradually decreased. More specifically, in patients at baseline and with accrued RFS of 1 and 2 years, the cRFS3 rates of the ACT group were 70.2%, 76.4%, and 88.1%, respectively, which were significantly higher than those of the OBS group (all *p*˂0.05). However, in patients with accrued RFS of 3 or more years, cRFS3 was comparable between the ACT and OBS groups (*p* > 0.05) (Fig. 2B). The actual 3-year RMST was significantly higher in the ACT group than that in the OBS group with a △RMST of 2.05 months (95% CI, 0.51–3.59, *p* = 0.009) at baseline. Similarly, in patients with accrued RFS of 1 and 2 years, the 3-year RMSTs of the ACT group were superior to that of the OBS group (*p*˂0.05). However, no significant differences in 3-year RMST were observed between the ACT and OBS groups in patients with accrued RFS of 3 or more years (*p* > 0.05) (Fig. 2C). A similar trend can be observed for the 5-year actual RFS, cRFS5, and 5-year RMST of the ACT and OBS groups (Fig. 2D-F). More details about △RMST between the ACT and OBS groups are shown in S-Table 5.

#### Timing and patterns of disease recurrence

**Fig. 3:**
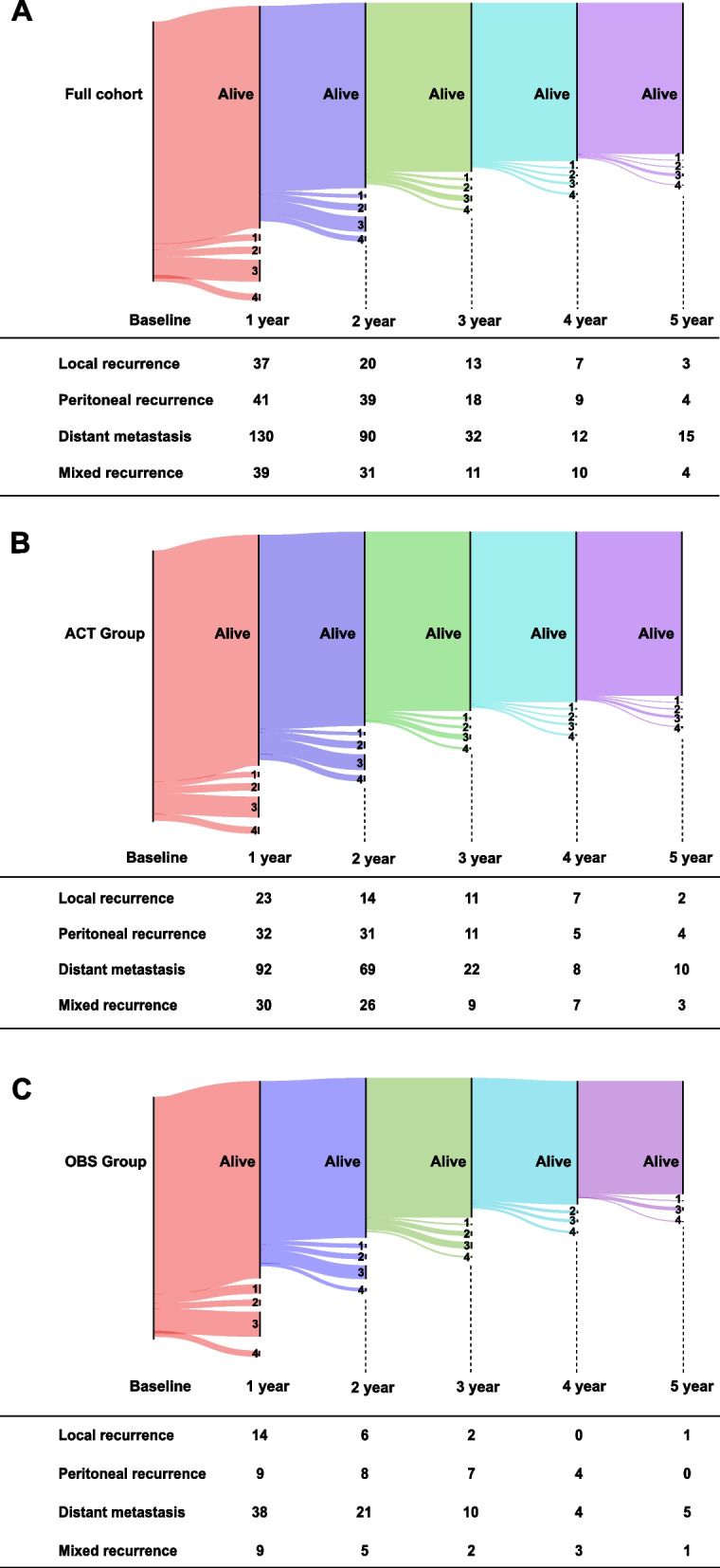
The Sankey diagram shows the dynamic transition from alive without recurrence to different recurrence types in the full cohort (**A**), the ACT group (**B**), and the OBS group (**C**) from baseline to 5 years after surgery. Notes: Patients with unknown recurrence site were excluded. 1:local recurrence; 2: peritoneal recurrence; 3: distant metastasis; 4: mixed recurrence. The table showed the number of patients at different time points

Intuitively, Fig. 3 shows the dynamic transition from alive without recurrence to different recurrence types (including local, peritoneal, distant metastasis, and mixed recurrence) in the full cohort (Fig. 3A), ACT group (Fig. 3B), and OBS group (Fig. 3C) from baseline to 5 years after surgery. For instance, there were 1,661 patients in the full cohort at baseline. After 1 year from surgery, local recurrence, peritoneal recurrence, distant metastasis, and mixed recurrence occurred in 37, 41, 130, and 39 patients, respectively. Likewise, recurrence may occur in the remaining patients with RFS from the next year until 5 years after surgery (Fig. 3A).

**Fig. 4:**
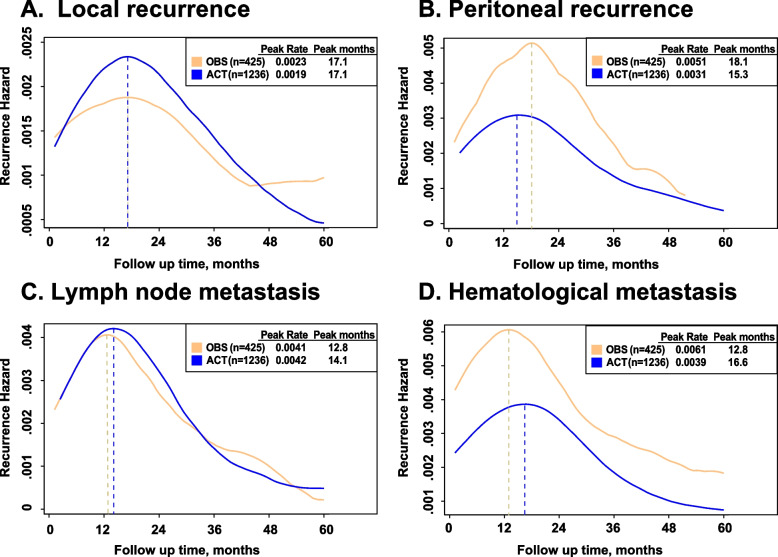
Comparison of dynamic recurrence hazard between the ACT and OBS groups stratified by different recurrence types

Figure 4 shows the smoothed hazard curves for the ACT and OBS groups stratified by recurrence types. Although the local recurrence hazard of the ACT group was slightly higher than that of the OBS group with the same peak time (17.1 months) in the early period, the trends reversed after 4 years from surgery (Fig. 4A). In terms of peritoneal recurrence (Fig. 4B), the hazard of the OBS group increased rapidly and decreased sharply. The OBS group demonstrated a higher peak rate (0.0051 vs. 0.0031) and later peak time (18.1 months vs. 15.3 months) than the ACT group. Regarding distant metastasis, dynamic hazard and peak time of lymph node metastasis were similar between the ACT and OBS groups (Fig. 4C). In contrast, the OBS group had a higher peak hazard (0.0061 vs. 0.0039) and earlier peak time (12.8 months vs. 16.6 months) in terms of hematological metastasis (Fig. 4D).

**Fig. 5:**
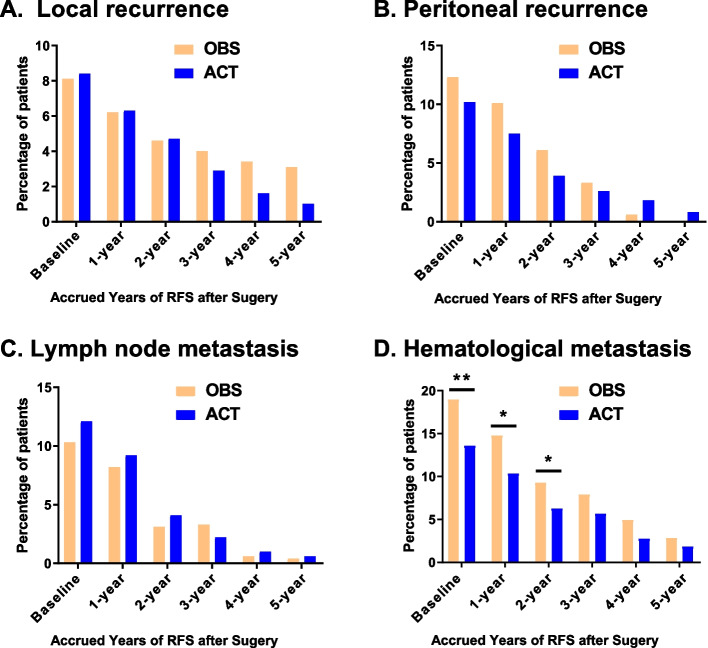
Comparison of adjusted cumulative recurrence rates between the ACT and OBS groups stratified by different recurrence types. * indicates *P* ˂ 0.05

Figure 5 demonstrates the incidences of different recurrence types in the ACT and OBS groups after IPTW adjustment. Overall, the hematological metastasis rate of the ACT group was lower than that of the OBS group at baseline (13.6% vs. 19.0%, *p* = 0.007). Likewise, in the subgroups of patients with accrued RFS of 1 and 2 years, hematological metastasis rates of the ACT group were significantly lower than those of the OBS group (10.4% vs. 14.8% and 6.3% vs. 9.3%, respectively; *p* < 0.05) (Fig. 5D). In terms of other recurrence types, the recurrence rates of both groups were comparable at any time point (*p* > 0.05) (Fig. 5A-C).

#### Dynamic changes in risk factors for disease recurrence

Time-dependent multivariate Cox analysis (Table 1) showed that ACT, pT stage, pN stage, and tumor size were independent prognostic factors for RFS at baseline. After 1 and 2 years of RFS, ACT was still an independent prognostic factor for RFS (p < 0.05). However, after 3 years of RFS, ACT lost its significance in the Cox regression (HR, 0.64; 95% CI, 0.38–1.08; *p* = 0.097). Notably, after 4 years of RFS, only pN stage independently affected RFS in patients with LAGC.

## Discussion

In recent years, several large-scale randomized controlled trials from Asia confirmed that ACT could significantly improve survival and recurrence rates in patients with LAGC [3, 5]. The ACTS-GC trial [3] implied that S-1 (oral fluoropyrimidine) monotherapy could achieve better 5-year overall survival (OS) and RFS rates than surgery alone (71.7% vs. 61.1%; HR, 0.67; 95% CI, 0.54–0.83; and 65.4% vs. 53.1%; HR, 0.65; 95% CI, 0.54–0.79, respectively). Subsequently, the CLASSIC trial [5] demonstrated that the XELOX regimen (capecitabine plus oxaliplatin), which was preferred in the European population, could also improve the 5-year disease-free survival and OS of Asian patients. Therefore, ACT has been considered the standard therapy for LAGC in Asia.

Since then, many well-designed studies have been carried out to explore the indications of ACT. Cheong et al. [6] constructed a single patient classifier (based on four genes), which effectively predicted the benefits of ACT. Choi et al. [7] also reported that microsatellite stable status (MSS) and programmed death ligand-1 (PD-L1) expressions were closely associated with the benefits of ACT. In addition, Sohn et al. [8] found that patients with chromosome instability (CIN) subtypes benefited most from ACT (HR, 0.39; 95% CI, 0.16–0.94;* P* = 0.03), while those with genetic stability benefited the least (HR, 0.83; 95% CI, 0.36–1.89; *P* = 0.65). However, the above models did not consider the long-term dynamic effects of ACT, which may lead to inaccurate estimation. With more patients surviving beyond 3 years, conditional survival analysis may be more appropriate for those who have already accrued RFS for a few years after surgery. Thus, our study explored the long-term dynamics of recurrence hazard in the ACT and OBS groups through IPTW-adjusted conditional survival analyses.

This study showed that although cRFS rates gradually increased with RFS already accrued, the differences in RMST gradually decreased. In patients at baseline or with accrued RFS of 1 and 2 years, the cRFS rates of the ACT group were significantly higher than those of the OBS group. However, after 3 years of RFS, the cRFS rates between both groups became comparable, and ACT lost its significance in the multivariate analysis. On one hand, these results are likely associated with features of tumor recurrence. Most disease recurrences occurred within 3 years after surgery. The recurrence hazards of the ACT and OBS groups significantly decreased over time thereafter, decreasing the differences in recurrence hazard between these two groups [31–33]. Moreover, the dynamic transition of recurrence patterns in the survivors of LAGC was consistent with the “natural selection effect” hypothesis of Zamboni et al. [34]. The hypothesis implies that most cases of high-risk LAGC recur soon after surgery, promoting the natural selection of lower-risk disease, and leading to more favorable prognosis in the survivors. ACT is usually completed within the first year after surgery [3, 5]. As drug concentration gradually decreases through metabolism, the suppression effects on tumor proliferation progressively diminish. On the other hand, our findings implied that more intensive surveillance should be considered in the patients with LAGC who did not receive ACT for some reasons (e.g., economic hardship and chemotherapeutic intolerance) within the first 2 years. After RFS of 3 years, the relationship between the ACT and OBS groups should be reassessed, according to cRFS. The surveillance strategy of the OBS group can be adjusted to the same as that of the ACT group to avoid wasting medical resources because the recurrence hazards were similar between these two groups. Meanwhile, during the follow-up, it is suggested that supervising doctors help patients understand cRFS and encourage them never to give up hope, which would enhance patients’ confidence in living and subsequently improve their quality of life, especially for those who didn’t receive ACT at the beginning.

Consistent with previous studies [3, 5], this study also suggested that dynamic changes in recurrence rates between the ACT and OBS groups may be associated with hematological metastasis, reflecting the importance of imaging examinations, such as thoracic and abdominal CT scanning or ultrasonography, for timely detection of organ metastasis in the early follow-up. Although the mechanism of ACT on cancer recurrence remains unclear, a previous study showed a close association between drug effect and hemodynamics, contributing to the eradication of micrometastatic tumor cells in the blood [35]. Thus, the effects of ACT tend to be more pronounced in hematological metastasis. In addition, the tumor immune microenvironment plays an important role in chemotherapeutic sensitivity [36–39]. With the impact of immune factors, the proliferation activity of gastric cancer cells metastasized to the specific organs may be more easily regulated by fluorouracil and platinum drugs, thereby limiting the further growth of tumor cells.

The present study had several limitations. First, our results were derived from an Eastern high-volume center and must be validated in the Western population. Second, although IPTW was used to minimize bias in clinicopathological characteristics between two groups, selection bias was inevitable, given the retrospective design. Additionally, chemotherapy cycles and regimens were not completely uniform. Lastly, because ACT was still the standard therapy in Asia, patients who received neoadjuvant chemotherapy were excluded to reduce the bias of results. Despite so, to the best of our knowledge, this study is the first well-designed IPTW-adjusted cohort study comparing the long-term dynamic changes of recurrence risk between the ACT and OBS groups of patients with LAGC.

## Conclusions

cRFS is a more accurate prognostic indicator in evaluating dynamic recurrence hazards. Although the survival benefits of ACT have been confirmed in numerous studies, the long-term dynamics of recurrence hazards should be of concern. More intensive surveillance for hematological metastasis should be considered in the OBS group within the first 2 years after surgery. After 3 years of RFS, it is necessary to reassess the dynamic recurrence hazards to optimize the existing surveillance strategy and alleviate patients’ anxiety. This study might provide effective references for clinical practice in the clinical setting of adjuvant chemotherapy.

### Supplementary Information

**Additional file 1.** 

**Additional file 2.** 

**Additional file 3.** 

## Data Availability

The datasets used and/or analyzed during the current study are available from the corresponding author on reasonable request.
