# Supplementary figures and images for: Evaluation of dynamic recurrence risk for locally advanced gastric cancer in the clinical setting of adjuvant chemotherapy: a real-world study with IPTW-based conditional recurrence analysis
Source: BMC Cancer. 2023 Oct 12;23:964. doi: 10.1186/s12885-023-11143-3 (PMC10568928; doi:10.1186/s12885-023-11143-3)

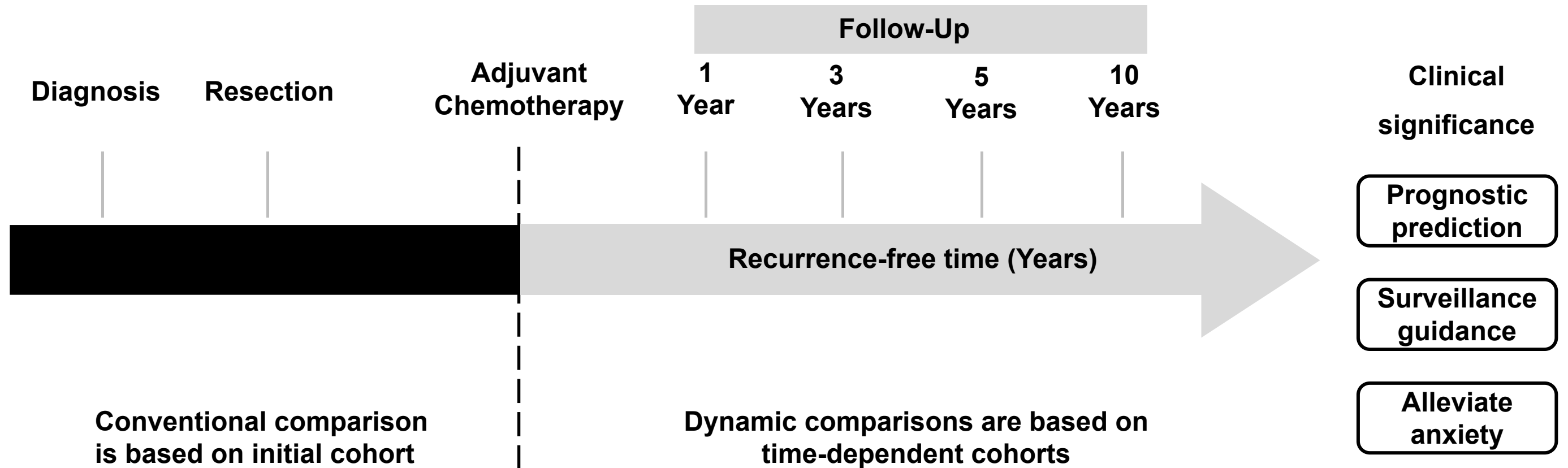

**S-Fig 1. Sketch map**

Supplement: Supplementary file 1 — Additional file 1. [file 12885_2023_11143_MOESM1_ESM.pdf]

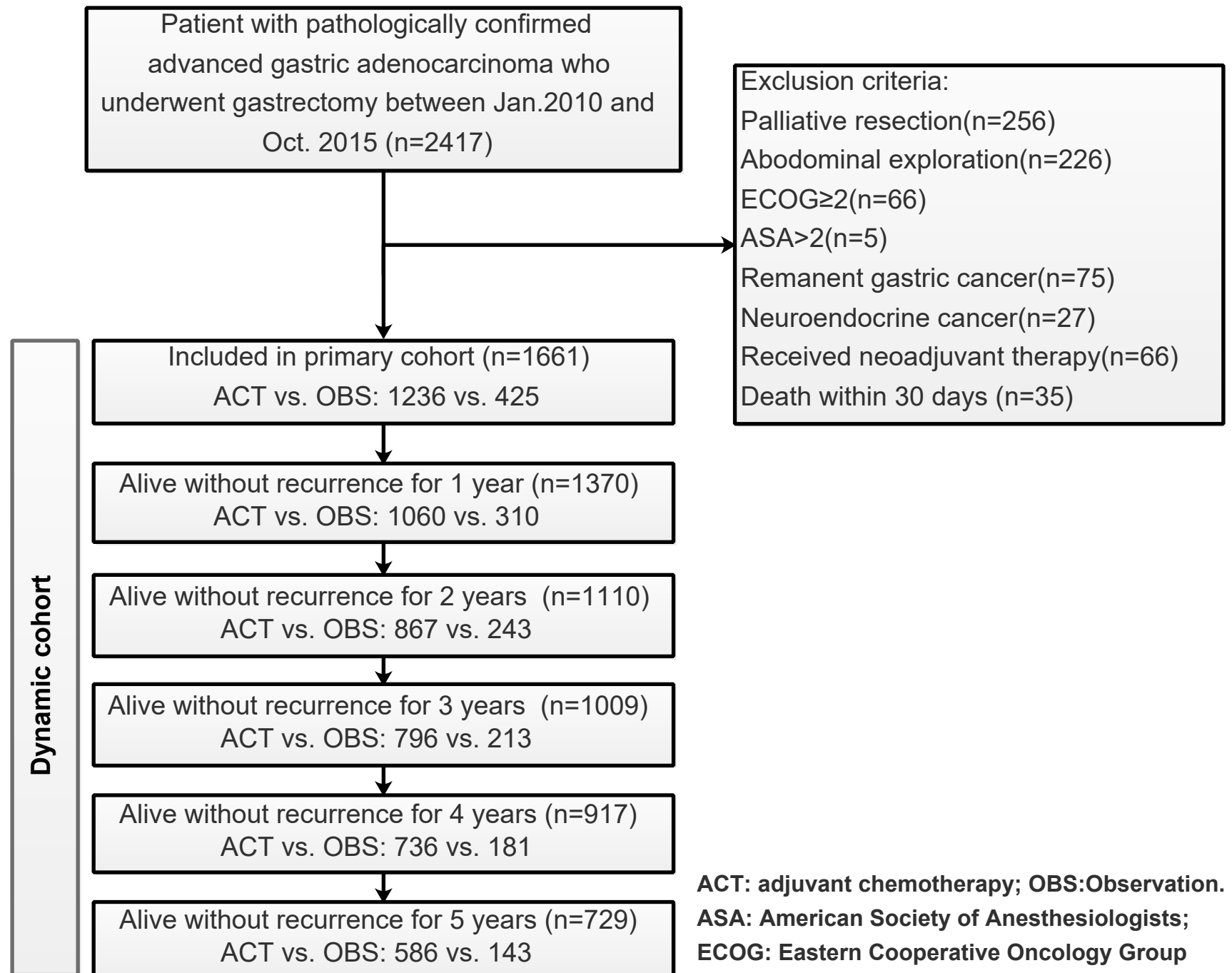

**S-Fig 2 Flow chart**

Supplement: Supplementary file 2 — Additional file 2. [file 12885_2023_11143_MOESM2_ESM.pdf]
