# Supplementary material for: Evaluation of dynamic recurrence risk for locally advanced gastric cancer in the clinical setting of adjuvant chemotherapy: a real-world study with IPTW-based conditional recurrence analysis
Source: BMC Cancer. 2023 Oct 12;23:964. doi: 10.1186/s12885-023-11143-3 (PMC10568928; doi:10.1186/s12885-023-11143-3)
Supplement: Supplementary file 3 — Additional file 3. [file 12885_2023_11143_MOESM3_ESM.docx]

**Evaluation of Dynamic Recurrence Risk for Locally Advanced Gastric Cancer in the clinical setting of adjuvant chemotherapy:**

***A real-world study with IPTW-based conditional recurrence analysis***

**Supplementary materials**

**S-Table 1.** Baseline characteristics.

**S-Table 2.** Conditional Probabilities of Recurrence-free Survival at Various Time Points in LAGC patients.

**S-Table 3.** Time-dependent univariate analysis of the Recurrence-free Survival for LAGC patients.

**S-Table 4.** Conditional Probabilities of Recurrence-free Survival at Various Time Points between the ACT and OBS groups.

**S-Table 5.** △RMST between the ACT and OBS groups.

**S-Figure 1.** Sketch map depicts the concept and clinical significance of conditional probability of recurrence-free survival.

**S-Figure 2.** Study flow chart.

**S-Table 1.** Baseline characteristics.

| **Clinical characteristics** | **Full cohort (n=1661)** |
| --- | --- |
| **Age n (%)** |  |
| <65 years | 1029 (62.0%) |
| ≥65 years | 632 (38.0%) |
| **BMI n (%)** |  |
| <25 kg/m^2^ | 1228 (73.9%) |
| ≥25 kg/m^2^ | 390 (23.5%) |
| unknown | 43 (2.6%) |
| **Sex n (%)** |  |
| Male | 1230 (74.1%) |
| Female | 431 (25.9%) |
| **Comorbidity n (%)** |  |
| No | 1200 (72.2%) |
| yes | 461 (27.8%) |
| **ASA n (%)** |  |
| Ⅰ | 945 (56.9%) |
| Ⅱ | 716 (43.1%) |
| **ECOG scores n (%)** |  |
| 0 | 208 (12.5%) |
| 1 | 1453 (87.5%) |
| **Oncological characteristics** |  |
| **Histology n (%)** |  |
| High/ moderate differentiated | 584 (35.2%) |
| low differentiated/ undifferentiated | 1056 (63.6%) |
| G_x_ | 21 (1.3%) |
| **Pathologic T Stage n (%)** |  |
| T1 | 37 (2.2%) |
| T2 | 133 (8.0%) |
| T3 | 730 (43.9%) |
| T4a | 761 (45.8%) |
| **Pathologic N Stage n (%)** |  |
| N0 | 255 (15.4%) |
| N1 | 283 (17.0%) |
| N2 | 391 (23.5%) |
| N3a | 478 (28.8%) |
| N3b | 254 (15.3%) |
| **Pathological TNM Stage of 8th AJCC n (%)** |  |
| Ⅱa | 264 (15.9%) |
| Ⅱb | 268 (16.1%) |
| Ⅲa | 416 (25.0%) |
| Ⅲb | 450 (27.1%) |
| Ⅲc | 263 (15.8%) |
| **Tumor Location n (%)** |  |
| Lower | 639 (38.5%) |
| Middle | 351 (21.1%) |
| Upper | 430 (25.9%) |
| Mixed | 241 (14.5%) |
| **Tumor Size n (%)** |  |
| <50 mm | 751 (45.2%) |
| ≥50 mm | 910 (54.8%) |
| **PNI n (%)** |  |
| No | 1157 (69.7%) |
| Yes | 504 (30.3%) |
| **LVI n (%)** |  |
| No | 1074 (64.7%) |
| Yes | 587 (35.3%) |
| **Other characteristics** |  |
| **Surgical approach n (%)** |  |
| OG | 284 (17.1%) |
| LG | 1377 (82.9%) |
| **The number of examined lymph nodes (n)** | 36.9 ± 14.2 |
| **Postoperative complication n (%)** |  |
| ˂III | 1234 (74.3%) |
| ≥III | 423 (25.5%) |
| Unknown | 4 (0.2%) |

**PNI: Perineural invasion; LVI: Lymphovascular invasion; ASA: American Society of Anesthesiologists; ECOG: Eastern Cooperative Oncology Group**

**S-Table 2.** Conditional Probabilities of Recurrence-free Survival at Various Time Points in LAGC patients.

|  | **Years of accrued Recurrence-free Survival (Yrs)** | | | | | | |
| --- | --- | --- | --- | --- | --- | --- | --- |
|  |  | **Actuarial Survival** | **1** | **2** | **3** | **4** | **5 Yrs** |
| **Recurrence-free Survival, (Yrs)** | | **n=1661** | **n=1370** | **n=1110** | **n=1009** | **n=917** | **n=729** |
| 1 | | 84.4% |  |  |  |  |  |
| 2 | | 72.3% | 85.7% |  |  |  |  |
| 3 | | 66.8% | 79.1% | 92.4% |  |  |  |
| 4 | | 64.1% | 76.0% | 88.7% | 96.0% |  |  |
| 5 | | 62.0% | 73.5% | 85.8% | 92.9% | 96.7% |  |
| 6 | | 60.9% | 72.2% | 84.3% | 91.2% | 95.0% | 98.2% |
| 7 | | 60.1% | 71.2% | 83.1% | 89.9% | 93.7% | 96.9% |
| 8 | | 59.2% | 70.1% | 81.8% | 88.5% | 92.2% | 95.4% |
| 9 | | 57.6% | 68.2% | 79.6% | 86.2% | 89.8% | 92.9% |
| 10 | | 57.6% | 68.2% | 79.6% | 86.2% | 89.8% | 92.9% |

**S-Table 3.** Time-dependent univariate analysis of the Recurrence-free Survival for LAGC patients.

| **Clinical characteristics** | **Baseline** | | | **1-year** | | | **2-year** | | | **3-year** | | | **4-year** | | | **5-year** | | |
| --- | --- | --- | --- | --- | --- | --- | --- | --- | --- | --- | --- | --- | --- | --- | --- | --- | --- | --- |
|  | **N=1661, E=605** | | | **N=1370, E=388** | | | **N=1110, E=176** | | | **N=1009, E=106** | | | **N=917, E=62** | | | **N=729, E=30** | | |
|  | **HR** | **95% CI** | **p value** | **HR** | **95% CI** | **p value** | **HR** | **95% CI** | **p value** | **HR** | **95% CI** | **p value** | **HR** | **95% CI** | **p value** | **HR** | **95% CI** | **p value** |
| **Age** |  |  |  |  |  |  |  |  |  |  |  |  |  |  |  |  |  |  |
| <65 years | Ref |  |  | Ref |  |  | Ref |  |  | Ref |  |  | Ref |  |  | Ref |  |  |
| ≥65 years | 1.2 | 1.02-1.42 | **0.025** | 1.25 | 1.01-1.54 | **0.041** | 1.27 | 0.93-1.73 | 0.137 | 1.31 | 0.88-1.96 | 0.189 | 1.04 | 0.61-1.78 | 0.888 | 1.12 | 0.50-2.52 | 0.777 |
| **BMI** |  |  |  |  |  |  |  |  |  |  |  |  |  |  |  |  |  |  |
| <25 kg/m2 | Ref |  |  | Ref |  |  | Ref |  |  | Ref |  |  | Ref |  |  | Ref |  |  |
| ≥25 kg/m2 | 0.91 | 0.75-1.11 | 0.361 | 0.98 | 0.77-1.26 | 0.894 | 0.88 | 0.60-1.27 | 0.482 | 1.02 | 0.64-1.62 | 0.935 | 0.99 | 0.54-1.81 | 0.973 | 0.97 | 0.39-2.43 | 0.946 |
| **Sex** |  |  |  |  |  |  |  |  |  |  |  |  |  |  |  |  |  |  |
| Male | Ref |  |  | Ref |  |  | Ref |  |  | Ref |  |  | Ref |  |  | Ref |  |  |
| Female | 1.02 | 0.85-1.22 | 0.834 | 1.08 | 0.85-1.37 | 0.526 | 0.93 | 0.65-1.33 | 0.692 | 1.1 | 0.70-1.72 | 0.687 | 0.72 | 0.37-1.38 | 0.317 | 0.82 | 0.31-2.18 | 0.692 |
| **Comorbidity** |  |  |  |  |  |  |  |  |  |  |  |  |  |  |  |  |  |  |
| No | Ref |  |  | Ref |  |  | Ref |  |  | Ref |  |  | Ref |  |  | Ref |  |  |
| yes | 1.03 | 0.86-1.24 | 0.717 | 1.11 | 0.88-1.40 | 0.378 | 0.95 | 0.67-1.35 | 0.783 | 0.84 | 0.52-1.35 | 0.472 | 0.83 | 0.45-1.54 | 0.562 | 1.12 | 0.47-2.67 | 0.795 |
| **ASA** |  |  |  |  |  |  |  |  |  |  |  |  |  |  |  |  |  |  |
| Ⅰ | Ref |  |  | Ref |  |  | Ref |  |  | Ref |  |  | Ref |  |  | Ref |  |  |
| Ⅱ | 1.09 | 0.92-1.28 | 0.317 | 1.12 | 0.91-1.38 | 0.296 | 0.9 | 0.66-1.24 | 0.525 | 0.84 | 0.55-1.27 | 0.400 | 0.98 | 0.58-1.67 | 0.939 | 1.02 | 0.46-2.29 | 0.959 |
| **ECOG scores** |  |  |  |  |  |  |  |  |  |  |  |  |  |  |  |  |  |  |
| 0 | Ref |  |  | Ref |  |  | Ref |  |  | Ref |  |  | Ref |  |  | Ref |  |  |
| 1 | 0.95 | 0.75-1.20 | 0.674 | 0.92 | 0.68-1.25 | 0.598 | 0.67 | 0.45-1.00 | 0.052 | 0.65 | 0.39-1.10 | 0.112 | 0.7 | 0.35-1.44 | 0.335 | 0.49 | 0.18-1.31 | 0.155 |
| **Histology** |  |  |  |  |  |  |  |  |  |  |  |  |  |  |  |  |  |  |
| High/ Moderate differentiated | Ref |  |  | Ref |  |  | Ref |  |  | Ref |  |  | Ref |  |  | Ref |  |  |
| Low differentiated/ Undifferentiated | 1.31 | 1.10-1.56 | **0.002** | 1.22 | 0.98-1.53 | 0.074 | 1.14 | 0.83-1.58 | 0.413 | 1.29 | 0.84-1.97 | 0.245 | 1.42 | 0.81-2.50 | 0.226 | 0.7 | 0.32-1.53 | 0.368 |
| **Pathologic T Stage** | | |  |  |  |  |  |  |  |  |  |  |  |  |  |  |  |  |
| T1 | Ref |  |  | Ref |  |  | Ref |  |  | Ref |  |  | Ref |  |  | Ref |  |  |
| T2 | 0.71 | 0.32-1.59 | 0.405 | 0.66 | 0.23-1.84 | 0.426 | 0.66 | 0.17-2.48 | 0.536 | 1.43 | 0.17-11.88 | 0.741 | 1.13 | 0.13-9.69 | 0.910 | 0.35 | 0.03-3.92 | 0.398 |
| T3 | 1.35 | 0.66-2.73 | 0.409 | 1.44 | 0.59-3.51 | 0.427 | 1.28 | 0.40-4.08 | 0.673 | 2.3 | 0.32-16.74 | 0.410 | 1.15 | 0.15-8.52 | 0.894 | 0.69 | 0.09-5.27 | 0.724 |
| T4a | 3 | 1.49-6.04 | **0.002** | 2.98 | 1.22-7.23 | **0.016** | 2.36 | 0.75-7.47 | 0.143 | 4.22 | 0.58-30.51 | 0.154 | 2.62 | 0.36-19.14 | 0.344 | 0.49 | 0.06-3.96 | 0.507 |
| **Pathologic N Stage** | | |  |  |  |  |  |  |  |  |  |  |  |  |  |  |  |  |
| N0 | Ref |  |  | Ref |  |  | Ref |  |  | Ref |  |  | Ref |  |  | Ref |  |  |
| N1 | 1.67 | 1.08-2.57 | **0.020** | 1.6 | 0.97-2.62 | 0.064 | 1.07 | 0.56-2.06 | 0.836 | 1.24 | 0.54-2.82 | 0.614 | 1.9 | 0.65-5.56 | 0.242 | 1.6 | 0.38-6.68 | 0.522 |
| N2 | 2.7 | 1.83-3.99 | **<0.001** | 2.55 | 1.63-3.99 | **<0.001** | 2.22 | 1.27-3.86 | **0.005** | 2.34 | 1.14-4.82 | **0.021** | 2.75 | 1.01-7.50 | **0.049** | 2.76 | 0.75-10.20 | 0.128 |
| N3a | 5.46 | 3.77-7.92 | **<0.001** | 4.48 | 2.91-6.90 | **<0.001** | 3.61 | 2.10-6.20 | **<0.001** | 4 | 1.98-8.09 | **<0.001** | 4.86 | 1.82-12.95 | **0.002** | 2.65 | 0.66-10.62 | 0.168 |
| N3b | 10.84 | 7.43-15.82 | **<0.001** | 8.59 | 5.49-13.45 | **<0.001** | 5.22 | 2.83-9.63 | **<0.001** | 4.67 | 2.05-10.67 | **<0.001** | 6.75 | 2.26-20.20 | **0.001** | 4.38 | 0.88-21.84 | 0.072 |
| **Tumor Location** | |  |  |  |  |  |  |  |  |  |  |  |  |  |  |  |  |  |
| Lower | Ref |  |  | Ref |  |  | Ref |  |  | Ref |  |  | Ref |  |  | Ref |  |  |
| Middle | 1.16 | 0.94-1.44 | 0.177 | 1.16 | 0.87-1.53 | 0.306 | 1.3 | 0.86-1.96 | 0.215 | 1.02 | 0.58-1.78 | 0.957 | 0.86 | 0.41-1.78 | 0.678 | 1.13 | 0.34-3.76 | 0.839 |
| Upper | 0.99 | 0.80-1.22 | 0.898 | 0.98 | 0.75-1.29 | 0.888 | 1.17 | 0.79-1.73 | 0.438 | 1.32 | 0.82-2.13 | 0.255 | 1.24 | 0.68-2.24 | 0.487 | 2.06 | 0.81-5.23 | 0.127 |
| Mixed | 1.61 | 1.28-2.02 | **<0.001** | 1.63 | 1.20-2.21 | **0.002** | 1.74 | 1.10-2.77 | **0.019** | 1.44 | 0.77-2.72 | 0.254 | 1.03 | 0.42-2.51 | 0.950 | 2.26 | 0.68-7.52 | 0.184 |
| **Tumor Size** |  |  |  |  |  |  |  |  |  |  |  |  |  |  |  |  |  |  |
| <50 mm | Ref |  |  | Ref |  |  | Ref |  |  | Ref |  |  | Ref |  |  | Ref |  |  |
| ≥50 mm | 1.98 | 1.67-2.34 | **<0.001** | 1.87 | 1.50-2.32 | **<0.001** | 1.61 | 1.18-2.19 | **0.003** | 1.64 | 1.10-2.44 | **0.015** | 1.84 | 1.10-3.08 | **0.021** | 1.64 | 0.75-3.57 | 0.212 |
| **PNI** |  |  |  |  |  |  |  |  |  |  |  |  |  |  |  |  |  |  |
| No | Ref |  |  | Ref |  |  | Ref |  |  | Ref |  |  | Ref |  |  | Ref |  |  |
| Yes | 1.45 | 1.23-1.72 | **<0.001** | 1.6 | 1.29-1.99 | **<0.001** | 1.33 | 0.95-1.85 | 0.095 | 1.55 | 1.01-2.36 | **0.043** | 1.51 | 0.87-2.62 | 0.147 | 1.51 | 0.63-3.60 | 0.353 |
| **LVI** |  |  |  |  |  |  |  |  |  |  |  |  |  |  |  |  |  |  |
| No | Ref |  |  | Ref |  |  | Ref |  |  | Ref |  |  | Ref |  |  | Ref |  |  |
| Yes | 1.64 | 1.40-1.93 | **<0.001** | 1.45 | 1.17-1.80 | **0.001** | 1.34 | 0.98-1.85 | 0.070 | 1.21 | 0.79-1.83 | 0.377 | 1.30 | 0.76-2.21 | 0.335 | 1.11 | 0.48-2.56 | 0.806 |
| **Surgical approach** | | |  |  |  |  |  |  |  |  |  |  |  |  |  |  |  |  |
| OG | Ref |  |  | Ref |  |  | Ref |  |  | Ref |  |  | Ref |  |  | Ref |  |  |
| LG | 0.81 | 0.66-0.99 | **0.040** | 0.71 | 0.55-0.92 | **0.009** | 0.7 | 0.48-1.02 | 0.065 | 0.78 | 0.47-1.30 | 0.342 | 0.88 | 0.45-1.74 | 0.718 | 0.98 | 0.34-2.84 | 0.966 |
| **The number of examined lymph nodes** | 1.01 | 1.00-1.01 | 0.054 | 1.01 | 1.00-1.02 | **0.007** | 1.01 | 1.00-1.02 | **0.014** | 1.01 | 0.99-1.02 | 0.293 | 1.00 | 0.98-1.02 | 0.987 | 1.00 | 0.97-1.03 | 0.823 |
| **Postoperative complication** | | | | | | |  |  |  |  |  |  |  |  |  |  |  |  |
| ˂III | Ref |  |  | Ref |  |  | Ref |  |  | Ref |  |  | Ref |  |  | Ref |  |  |
| ≥III | 1.65 | 1.14-2.40 | **0.008** | 1.51 | 0.88-2.57 | 0.134 | 1.48 | 0.65-3.34 | 0.348 | 0.88 | 0.22-3.59 | 0.863 | - | - | - | - | - | - |
| **Adjuvant Chemotherapy** | | |  |  |  |  |  |  |  |  |  |  |  |  |  |  |  |  |
| No | Ref |  |  | Ref |  |  | Ref |  |  | Ref |  |  | Ref |  |  | Ref |  |  |
| yes | 0.75 | 0.63-0.90 | **0.002** | 0.76 | 0.60-0.97 | **0.026** | 0.69 | 0.49-0.97 | **0.034** | 0.67 | 0.43-1.04 | 0.074 | 0.75 | 0.42-1.34 | 0.324 | 0.68 | 0.29-1.62 | 0.386 |

**PNI: Perineural invasion; LVI: Lymphovascular invasion; ASA: American Society of Anesthesiologists; ECOG: Eastern Cooperative Oncology Group**

**S-Table 4.** Conditional Probabilities of Recurrence-free Survival at Various Time Points between the ACT and OBS groups.

|  | **Years of accrued Recurrence-free Survival (Yrs)** | | | | | |
| --- | --- | --- | --- | --- | --- | --- |
|  | **Actuarial survival** | **1** | **2** | **3** | **4** | **5 Yrs** |
| **Recurrence-free Survival, (Yrs)** | **n=1661** | **n=1370** | **n=1110** | **n=1009** | **n=917** | **n=729** |
| **OBS vs ACT** | **425 vs 1236** | **310 vs 1060** | **243 vs 867** | **213 vs 796** | **181 vs 736** | **143 vs 586** |
| 1 | 83.8% vs 88.0% |  |  |  |  |  |
| 2 | 69.5% vs 74.0% | 82.9% vs 84.1% |  |  |  |  |
| 3 | 62.4% vs 70.2% | 74.5% vs 79.8% | 89.8% vs 94.9% |  |  |  |
| 4 | 58.0% vs 67.2% | 69.2% vs 76.4% | 83.5% vs 90.8% | 92.9% vs 95.7% |  |  |
| 5 | 55.8% vs 65.2% | 66.6% vs 74.1% | 80.3% vs 88.1% | 89.4% vs 92.9% | 96.2% vs 97.0% |  |
| 6 | 55.0% vs 63.9% | 65.6% vs 72.6% | 79.1% vs 86.4% | 88.1% vs 91.0% | 94.8% vs 95.1% | 98.6% vs 98.0% |
| 7 | 53.2% vs 62.8% | 63.5% vs 71.4% | 76.5% vs 84.9% | 85.3% vs 89.5% | 91.7% vs 93.5% | 95.3% vs 96.3% |
| 8 | 52.9% vs 61.8% | 63.1% vs 70.2% | 76.1% vs 83.5% | 84.8% vs 88.0% | 91.2% vs 92.0% | 94.8% vs 94.8% |
| 9 | 52.9% vs 61.8% | 63.1% vs 70.2% | 76.1% vs 83.5% | 84.8% vs 88.0% | 91.2% vs 92.0% | 94.8% vs 94.8% |
| 10 | 52.9% vs 61.8% | 63.1% vs 70.2% | 76.1% vs 83.5% | 84.8% vs 88.0% | 91.2% vs 92.0% | 94.8% vs 94.8% |

**ACT, adjuvant chemotherapy group; OBS, observational group.**

**S-Table 5.** △RMST between the ACT and OBS groups.

|  |  | **RMST difference of Recurrence-free Survival for Patients Surviving (Yrs) between ACT and OBS group** | | | | |
| --- | --- | --- | --- | --- | --- | --- |
|  | **Actuarial Survival** | **Survived 1-year** | **Survived 2-year** | **Survived 3-year** | **Survived 4-year** | **Survived 5-year** |
| **Recurrence-free Survival,(Yrs)** | **n=1661** | **n=1370** | **n=1110** | **n=1009** | **n=917** | **n=729** |
| **OBS vs ACT** | **425 vs 1236** | **300 vs 1032** | **237 vs 862** | **208 vs 797** | **179 vs 734** | **142 vs 564** |
| 1 | **0.37 (0.06-0.68)** |  |  |  |  |  |
| 2 | **1.15 (0.26-2.03)** | 0.39 (-0.1-0.88) |  |  |  |  |
| 3 | **2.05 (0.51-3.59)** | 0.98 (-0.15-2.11) | **0.53 (0.11-0.95)** |  |  |  |
| 4 | **3.10 (0.86-5.33)** | 1.77 (-0.06-3.61) | **1.32 (0.31-2.33)** | 0.24 (-0.13-0.61) |  |  |
| 5 | **4.26 (1.3-7.21)** | 2.71 (0.13-5.29) | **2.29 (0.61-3.96)** | 0.70 (-0.21-1.6) | 0.12 (-0.19-0.44) |  |
| 6 | **5.34 (1.65-9.04)** | **3.57 (0.22-6.93)** | **3.16 (0.77-5.56)** | 1.07 (-0.44-2.59) | 0.17 (-0.59-0.93) | -0.04 (-0.26-0.18) |
| 7 | **6.55 (2.11-11)** | **4.59 (0.45-8.73)** | **4.23 (1.07-7.39)** | 1.67 (-0.53-3.86) | 0.45(-0.88-1.78) | 0.17 (-0.52-0.86) |
| 8 | **7.67 (2.46-12.89)** | **5.51 (0.54-10.47)** | **5.18 (1.21-9.15)** | 2.14 (-0.8-5.09) | 0.62 (-1.38-2.61) | 0.25 (-1.03-1.53) |
| 9 | **8.72 (2.72-14.73)** | **6.35 (0.54-12.15)** | **6.04 (1.22-10.85)** | 2.53 (-1.22-6.28) | 0.69 (-2.04-3.41) | 0.24 (-1.71-2.2) |
| 10 | **9.87 (3.04-16.7)** | **7.31 (0.6-14.02)** | **7.05 (1.28-12.82)** | 3.09 (-1.62-7.8) | 0.95 (-2.74-4.64) | 0.43 (-2.5-3.37) |

**Bold font indicates statistical significance (p<0.05). ACT, adjuvant chemotherapy group; OBS, observational group.**
